# Inguinal hernia in southern Brazil - challenges in follow-up and recurrence rates

**DOI:** 10.1590/0100-6991e-20223238-en

**Published:** 2022-08-25

**Authors:** RODRIGO PILTCHER-DA-SILVA, DEBORA OLIVEIRA HÜTTEN, ARTUR GEHRES TRAPP, PEDRO SAN MARTIN SOARES, TIAGO LIMA CASTRO, SIMONI BOHNENBERGER, EDUARDO CASTELLI KROTH, JORGE ARMANDO REYES PINTO, CAROLINE GREHS, DANIELLE CRISTINA TOMASI, LEANDRO TOTTI CAVAZZOLA

**Affiliations:** 1 - Hospital de Clínicas de Porto Alegre, General Surgery Service - Porto Alegre - RS - Brasil; 2 - Universidade Federal de Pelotas, Public Health Department - Pelotas - RS - Brasil

**Keywords:** Hernia, Inguinal Hernia, Groin Hernia, Recurrent Hernia, Hernia, Hernia Ingunial, Hernia Femoral, Recidiva

## Abstract

**Introduction:**

recurrence rates for primary hernia repair range from 0.5 to 15 percent depending upon the hernia site, type of repair, and clinical circumstances. Many risk factors are known and they must be considered before the procedure. In developing countries, follow up and maintenance of databases are critical to understand the real numbers.

**Methods:**

a retrospective cohort study analyzed adult patients who have undergone inguinal hernia repair at Hospital de Clínicas de Porto Alegre, a tertiary care government public hospital, between 2013 and 2015. Medical records, telephone, and letter contact have been reviewed in order to complete the minimum period of 5 years of follow-up. The analyzed data focused on the surgeon’s experience and the recurrence rate in 5 years of follow-up.

**Results:**

a total of 1094 medical records were selected and a complete five years follow-up were possible in 454 patients - 538 inguinal hernia repairs due to bilateral approach in 84 patients. These 454 patients answered, in a validated questionnaire about symptoms of recurrence. The total recurrence rate was 9.29%. For the patients who had Nyhus IV, recurrence rate was 24.1% against 9.9% after primary hernia repair, with a 2.4 higher risk. There was no difference in recurrence between surgeons and training surgeons.

**Conclusion:**

our data reveal an acceptable recurrence rate in a tertiary care hospital with residents, and to our knowledge is the first Brazilian report with long term follow up. An increased re-recurrent hernia was found when compared with primary hernia repair.

## RESUMO

**Introdução:**

a recorrência da hérnia inguinal após hernioplastia varia de 0,5 a 15 por cento, dependendo do local da hérnia, tipo de reparo e circunstâncias clínicas. Muitos fatores de risco são conhecidos e devem ser considerados antes do procedimento. Acompanhamento e adequado bancos de dados são fundamentais para entender a incidência de recidiva.

**Métodos:**

estudo de coorte retrospectivo analisou hernioplastias inguinais realizados no Hospital de Clínicas de Porto Alegre entre 2013 e 2015. Para concluir 5 anos de seguimento, analisamos o prontuário e fizemos contato telefônico e por correio.

**Resultados:**

o total de 1094 registros médicos foram selecionados e um seguimento de pelo menos 5 anos foi possível em 454 pacientes - 538 reparos de hérnia inguinal devido à abordagem bilateral em 84 pacientes. Os pacientes responderam um questionário validado sobre sintomas de recorrência. A taxa total de recorrência foi de 9,29%. No grupo masculino, a recorrência foi de 10% contra 4% no grupo feminino. Para os pacientes com hérnia Nyhus IV, a recidiva foi de 24% contra 8% após o reparo da hérnia primária, com um risco de 2,8 maior. Não houve diferença na recorrência entre cirurgiões experientes e em treinamento.

**Conclusão:**

nossos dados revelam uma taxa de recorrência aceitável em um hospital de ensino, e para o nosso conhecimento é o primeiro artigo com acompanhamento de longo prazo no sul do Brasil. A re-recidiva da hérnia foi maior quando comparada com o reparo da hérnia primária.

## INTRODUCTION

Groin hernia repair remains one of the most common surgeries worldwide with more than 20 million surgeries being performed every year^1^ ^,^ ^2^. However, despite all the progress made in inguinal hernia surgery - development of meshes and laparoscopic surgery - the median recurrence rate can reach 15%, according to the hernia site, repair technique and other clinical conditions^2^ ^-^ ^4^. 

The recurrence may occur early after surgery or many years later, until 40 to 50 years after surgery^5^. Besides, there are many studies describing a follow-up for hernia recurrence of 5 years, and according to larger follow-up studies, just 40% of recurrences are diagnosed during this time^3^ ^,^ ^5^. 

Recurrence rates dropped significantly after the advent of the Lichtenstein and other mesh repair techniques, leaving no more benefit for elective repair of inguinal hernia without the use of mesh in the adult population. Laparoscopic techniques have emerged as a less invasive surgery and have also contributed to reduction of recurrence rates - transabdominal preperitoneal (TAPP) and totally extraperitoneal (TEP)^6^ ^,^ ^7^. Thus, the use of mesh and the surgeon’s expertise are crucial to obtain a good result^8^. Factors such as surgical technique, type of mesh, gender, family history, comorbidities, and type of hernia also can influence the risk of recurrence, and the risk is even greater if the repair is a recurrence or a second or third recurrence^3^ ^,^ ^4^. 

The aim of this study is to identify recurrence rates in 5 years of follow-up at a teaching and tertiary level of health care hospital in the south of Brazil. In addition to these recurrence rates, we expose the difficulty in concluding a long follow-up in research on benign surgical diseases, here using hernias as an example. These challenges have been recently exposed in a Letter to Editors to The World Journal of Hernia and Abdominal Wall Surgery^9^.

## METHODS

Retrospective review of medical records of patients undergoing laparoscopic or conventional groin hernia surgery at Hospital de Clínicas de Porto Alegre from 2013 until 2015. The following variables were analyzed: gender, age, comorbidities, surgeon, surgery technique, hernia Nyhus classification (Table 1), perioperative findings, and recurrence rates with a 5 year follow-up. All these variables were identified by medical record evaluation, except the recurrence rate that was achieved by phone and mail contact - for those without an up-to-date telephone record at the hospital, in the letter we asked for the new contact number and then applied the questionnaire by telephone. For identification of hernia recurrence during telephone contact we used the Ventral Hernia Recurrence Inventory (VHRI): with the following questions being - “Do you feel or see a bulge?”, “Do you feel your hernia has come back?” and “Have you undergone surgery for hernia recurrence in another hospital?”. 

**Table 1:** Nyhus classification.

I	Indirect hernia with normal deep inguinal ring
II	Indirect hernia with an enlarged deep inguinal ring. Intact posterior wall
IIIa	Direct hernia; posterior wall defect only
IIIb	Indirect hernia with enlargement of deep inguinal ring and posterior wall defect
IIIc	Femoral hernia
IVa	Recurrent direct hernia
IVb	Recurrent indirect hernia
IVc	Recurrent femoral hernia
IVd	Combination of IVa, IVb and IVc hernias.

Exclusion criterion was age below 15 years old at the time of the surgery and the impossibility of contact to conclude the follow-up.

For statistical analysis, Chi-Square and Mann-Whitney tests were used using Stata software version 15.0.

## RESULTS

We have selected 1094 medical records of patients that had undergone inguinal hernia repair between 2013 and 2015 in our institution. Since we had difficulties contacting all those patients for revaluation, we ended up with 454 patients with a follow-up of at least 5 years. 410 (90.3%) patients were male and the average age of the total patients was 59.6 years (±14.9 years). In the total sample, the most common comorbidity was systemic arterial hypertension, present in 41.4% of the patients. In addition, 32.8% of the patients had no comorbidities (Table 2). The overall recurrence rate was 9.3% (49 repairs). In female patients, we found a recurrence rate of 4.5% (95% CI 0.5% - 13.9%) while in male patients the recurrence rate was 11.5% (95% CI 7.6% - 13.8%), which is not statistically significant. In cases with recurrence, 10.1% had comorbidities against 12.6% who did not have, showing a difference without statistical significance (p-value: 0.455; Table 2). Surgeries were performed by medical residents in 67.5% (303 repairs) of the time (always under direct supervision of senior surgeons) and by general surgeons who had already graduated (general surgeons, residence preceptors) in 32.5% (146 repairs). An unexpected result was that there was no statistical difference between the recurrence rates of patients operated by training and experienced surgeons, 12.3% vs 10.2%, respectively.

**Table 2:** Baseline Patient Characteristics. Hospital de Clínicas de Porto Alegre. Porto Alegre, 2013 - 2015 (n=454).

	Hernia recurrence			
		No	Yes	
	n (%)	n (%)	n (%)	p-value
Gender				0,160^a^
Male	410 (90,3)	363 (88,5)	47 (11,5)	
Female	44 (9,7)	42 (95,5)	2 (4,5)	
Age - mean (SD)	59,6 (14,9)	59,74 (15,1)	58,22 (13,5)	0.466^b^
Comorbidities				0,455^a^
Yes	276 (67,2)	248 (89,9)	28 (10,1)	
No	135 (32,8)	118 (87,4)	17 (12,6)	
Smoking				0,350^a^
Yes	36 (8,8)	31 (86,1)	5 (13,9)	
No	375 (91,2)	335 (89,3)	40 (10,7)	
IRC				0,077^a^
Yes	6 (1,5)	4 (66,7)	2 (33,3)	
No	405 (98,5)	362 (89,4)	43 (10,6)	
Obesity				0,638^a^
Yes	35 (8,5)	32 (91,4)	3 (8,6)	
No	376 (91,5)	334 (88,8)	42 (11,2)	
DPOC				0,768^a^
Yes	12 (2,9)	11 (91,7)	1 (8,3)	
No	399 (97,1)	355 (89,0)	44 (11,0)	
Stroke				0,987^a^
Yes	9 (2,2)	8 (88,9)	1 (11,1)	
No	402 (97,8)	358 (89,1)	44 (10,9)	
Malignant disease				0,495^a^
Yes	76 (18,5)	66 (86,8)	10 (13,2)	
No	335 (81,5)	300 (89,6)	35 (10,4)	
SH				0,246^a^
Yes	170 (41,4)	155 (91,2)	15 (8,8)	
No	241 (58,6)	211 (87,6)	30 (12,4)	
DM				0,694^a^
Yes	39 (9,5)	34 (87,2)	5 (12,8)	
No	372 (90,5)	332 (89,2)	40 (10,8)	
HF				0,841^a^
Yes	11 (2,7)	10 (90,9)	1 (9,1)	
No	400 (97,3)	356 (89,0)	44 (11,0)	
Surgeon				0,504^a^
General Surgeon	146 (32,5)	128 (87,7)	18 (12,3)	
General Surgery resident	303 (67,5)	272 (89,8)	31 (10,2)	
Nyhus				0,106^a^
I and II	189 (41,8)	175 (92,7)	14 (7,3)	
III	265 (58,2)	141 (87,9)	114 (12,5)	
Recurrence vs re-recurrence rates (Nyhus)				0,018^a^
I-III	422 (93,6)	384 (91,0)	38 (9,9)	
IV	24 (6,4)	18 (75,9)	6 (24,1)	

HAS: hipertensão sistêmica; DM: diabetes mellitus; DPOC: doença pulmonar obstrutiva crônica; ICC: insuficiência cardíaca; a: teste quiquadrado; b: Teste de Mann -Whitney.

**Figure 1:**
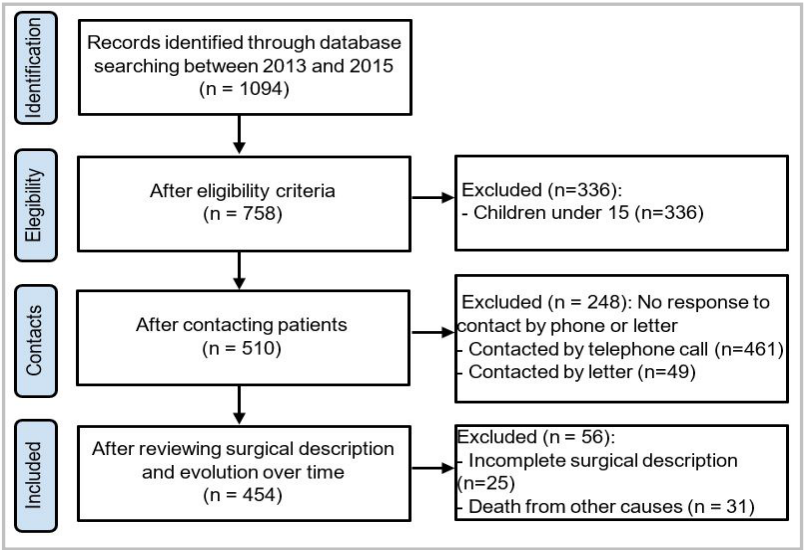
Database results.

Of these 454 patients, 84 underwent bilateral hernia repair, with 27 Lichtenstein technique, 55 transabdominal preperitoneal repair (TAPP), 1 Stoppa repair, and 1 totally extraperitoneal repair (TEP). So, considering bilateral correction as two different hernia repairs, due to the risk of recurrence on both sides, we ending with 538 inguinal hernia corrections. Among the 538 hernioplasties, the most common procedure was the Lichtenstein technique with 379 repairs (70.4%), followed by TAPP 145 repairs (26.9%), MacVay with mesh 7 repairs (1.3%), Stoppa 2 repairs (0.3%), Bassini 2 repairs (0.3%), TEP 2 repairs (0.3%) and MacVay 1 repair (0.1). All patients with posterior wall defect, including femoral hernia, were described and classified together according to the Nyhus classification.

Regarding the classification of the type of hernia, indirect hernias without posterior wall defect (Nyhus I and II,) represented 41.8% (189 repairs) of the total procedures with a recurrence rate of 7.3% (95% CI 3.9%-12.2%) while direct hernias (Nyhus IIIa-c) represented 58.2% (141 repairs) of the total procedures with a recurrence rate of 12.5% (95% CI 10.3% - 15.2%). We did not find statistical difference between these two groups (p-value: 0.106), however, it seems to have clinical significance due to the average number found. Another finding of this study was that the recurrent hernias (Nyhus IV) operated between 2013 and 2015 had a higher rate of recurrence (re-recurrence) when compared with the primary hernia repair (p-value: 0.034). The risk of re-recurrence of inguinal hernia was 2.4 times greater when a procedure was performed for recurrence.

## DISCUSSION

Despite the claim that hernia follow-up needs to be for a longer period of time, up to 40 to 50 years, in our reality, it is very difficult or even impossible, at this moment, to perform such a long term follow-up without any database or other mechanisms that allow prolonged contact with the patient. In 2020, we exposed, in a Letter to Hernia Journal, the difficulties in making a suitable follow-up of patients with benign diseases taking into consideration the reality of the national health system in Brazil, due to a large number of patients, oncology patients that need a closer follow-up by the same surgical team and the characteristics of the population^9^. It is known that long-term follow-up is not easy anywhere, but there are research tools that can help and the future looks promising, as was presented by our esteemed colleagues D. Cuccurulo, J. -F. Gillion, L. N. Jorgensen & H. Friis-Andersen, F. Köckerling, John Morrison, E. Perea del Pozo et al., citing the functioning of their hernia database in different countries around the world^10^ ^-^ ^15^. 

Currently, phone contact has been used as a tool to optimize patient follow-up and provide the development of a scientific medical study without significant loss in the sensitivity and specificity of the evaluation^16^. This has already been demonstrated by a study carried out in Brazil and the USA, where the sensitivity and specificity of the telephone evaluation for recurrence of ventral hernia, in Brazil, was 94% and 93%, respectively. In addition, the best performing question was “Do you feel or see a bulge?”, with Youden’s Index of 0.86. This study was carried out using the VHRI questionnaire^17^. As the symptoms and perception of ventral and inguinal hernia are similar, the validity of the method can be extrapolated to the evaluation carried out in this study.

The risk of developing groin hernia varies between genders, reaching values of up to 43% for men and only 6% for women^2^. The difference in risk of recurrence between men and women exposed in the above results, 11.5% vs 4.5% respectively, is also found in the literature, where the average recurrence in males varies between 11.3% to 14.3% against only 7% to 7.4% in females^18^. The reason for this difference is still unclear and probably related to lifestyle and work activity^18^ ^-^ ^20^. The only treatment available is surgical, which should be considered even in those oligosymptomatic and asymptomatic cases, since due to the progression of symptoms, 38% will need surgery within 3 years and 70% within 5 to 10 years if watchful waiting is the chosen approach^21^ ^-^ ^23^.

The presence of comorbidities such as diabetes unsettle the healing process and increase the risk of recurrence. Life habits, such as smoking, promote imbalance in the healing process and the composition of collagen, also influencing the appearance of hernias, by promoting the weakening of the abdominal wall and culminating in direct hernia^2^ ^,^ ^24^. Another factor that is believed to be related to the incidence of groin hernias and the recurrence after repair is the patient’s work activity; (this is believed for jobs that require great mechanical strength and therefore may cause an increase in intra-abdominal pressure). However, there is no consistent evidence to prove this hypothesis^19^ ^,^ ^25^.

A pilot study also carried out at the Hospital de Clínicas de Porto Alegre with analysis and follow-up of hernioplasties performed in 2006 had already identified an average age and ASA classification similar to that found in this study^26^. This data are also corroborated by the worldwide literature, which shows a similar average age and older age as a risk factor for developing inguinal hernia^2^ ^,^ ^4^ ^,^ ^27^.

The occurrence of recurrence in 5 years of follow-up identified in this study was 9.29% of patients. Although the follow-up was’t as long as we wanted, the value found is similar with others in the current literature, which varies between 10 and 15%^2^ ^-^ ^4^. Reference centers for inguinal hernia treatment are those with a large volume of surgeries a year, that is, more than 126 procedures a year, a group in which our institution fits^28^. The total number of procedures in the institution added to the number of surgeries performed during training and performed by senior surgeons a year, tend to promote positive reinforcement in the results, reducing the recurrence rate^8^ ^,^ ^28^.

The absence of a significant difference in recurrence between trainees and senior surgeons may be due to the constant presence of preceptorship in this institution. Besides, only the simplest cases are left for first-year residents, until they have the necessary learning curve. The learning curve for performing hernioplasty using the Lichtenstein technique varies in the literature between 25 and 40 procedures^29^ ^,^ ^30^. It has been observed that there is a greater relative risk of recurrence in cases where the surgeon performs a few surgeries a year (<10 procedures per year), regardless of training time^8^ ^,^ ^29^ ^,^ ^30^.

In addition, the most advanced techniques, also performed at the institution and evaluated in this article, need an even longer training time, since they are video laparoscopic techniques and, therefore, the surgeon must already have knowledge of anatomy and technical capacity for their execution. The learning curve for performing TAPP seems to be 50 to 100 procedures, while for TEP it varies from 100 to 250 procedures^29^ ^-^ ^32^. It is important to note that the two laparoscopic techniques, also with the use of mesh, maintained long-term recurrence rates similar to that of the Lichtenstein technique when compared to surgeons with experience in the area^33^ ^,^ ^34^.

It is essential to understand that there is an important pathological difference between the types of hernias, and those with the destruction of the posterior wall (Nyhus IIIa-c) represent greater severity and greater risk of perioperative complications and recurrence, often related to changes in collagen quality or in the relationship between type I and III collagen, whether due to genetics, comorbidities, or lifestyle^2^ ^,^ ^24^. This resulted in thinner collagen fibers and diminished mechanical strength. Meanwhile, indirect inguinal hernia is the maintenance of patency of the peritoneum-vaginal canal, without major anatomical destruction and often identified in youth. 

Another finding of this study was that the recurrent hernias (Nyhus IV) operated between 2013 and 2015 had a higher rate of recurrence (re-recurrence) when compared with the primary hernia repair, 24.1% vs 9.9%, respectively, and a 2.8 times higher risk of recurrence. Such data is corroborated by the literature, in which the repair of recurrences is associated with higher rates of complications and recurrences. In these cases, the laparoscopic repair is shown to be superior^18^ ^,^ ^35^.

The main limitation of the present study is the impossibility of long-term follow-up in a significant number of patients, approximately 50%. Due to the difficulty in face-to-face reassessment of patients, we used a previously validated questionnaire to assess hernia recurrence. Although validated in Brazil, it is not as accurate as face-to-face medical evaluation or imaging evaluation.

## CONCLUSION

The loss to follow-up of approximately 50% is significant, measures to improve follow-up should be evaluated. The use of questionnaires through the various means of communication came to assist in patient care and research, always having to be validated.

As demonstrated by our data with low rate of follow-up, we should work to promote the formation of a Unified hernia DataBase, which can be an important tool to achieve more important results. So far, our data demonstrate an incidence of groin hernia recurrence that it’s comparable to the current literature. It also shows that there is an increased risk of re-recurrence, 2.8 times greater. As discussed above, we found a higher rate of recurrence in the male group even without statistical significance, which may be clinically relevant and therefore further studies should be carried out. Thus, recurrent hernia has to be regarded as a multifactorial surgical complication and doctor and patient must be aware of it. 

This study demonstrates a retrospective analysis of a sample from an educational institution in the south of Brazil. Therefore clinical trials, with larger samples and with greater geographical diversification are necessary to corroborate the results.
